# A medico-legal approach to developing a structured decision-making process for patients refusing blood transfusions

**DOI:** 10.3389/fpubh.2025.1687101

**Published:** 2025-10-13

**Authors:** Lucia Tattoli, Davide Santovito

**Affiliations:** ^1^Section of Legal Medicine, Department of Public Health and Pediatric Sciences, University of Turin, Turin, Italy; ^2^Section of Legal Medicine, University Hospital Città della Salute e della Scienza di Torino, Turin, Italy

**Keywords:** informed consent, blood transfusion refusal, autonomy, patient blood management, risk management, medico-legal expert

## Abstract

**Introduction:**

The healthcare providers should respect patients’ autonomy in making treatment decisions, which affect their health and wellbeing. Refusing life-saving treatments like blood transfusions raises ethical and legal concerns that should be discussed in informed consent. The dilemma is between respecting patient’s autonomy and beneficence principle, the physician’s duty of care. This article explores the communication and information process to provide to adult patients who refuse blood transfusions or related blood products, focusing on legal frameworks, clinical practices, and medico-legal risk management.

**Methods:**

The authors conducted a retrospective analysis of medico-legal consultations on patients ‘refusal of blood and blood components transfusions in the Hospital “Città della Salute e della Scienza” of Turin (Italy) from 2017 to 2021. The Authors divided the consultation process into seven phases and 27 sub-phases which were analyzed through the PROSA risk assessment software. The strengths, weaknesses, opportunities, and threats (SWOT) analysis was used to evaluate the efficacy and potential of this structured protocol of consultation in clinical applications.

**Results:**

Based on 16 medico-legal consultations, high-risk sub-phases include medical record analysis, care pathway selection shared by the equipe; alternative therapy identification; communication regarding the transfusion and the Patient Blood Management process; communication regarding the not feasibility without transfusion. SWOT analysis showed that the informed consent process indicated advantages in 74% of aspects versus disadvantages in 26%.

**Discussion:**

Healthcare professionals should follow predictable, effective, efficient, transparent, and traceable guidelines when dealing with patients who refuse blood transfusions. A structured communication process, as a part of the informed consent, offers a replicable framework that can be adapted to other clinical contexts where high-stakes decision-making intersects with patient rights, supporting a culture of safety, transparency, and continuous improvement in healthcare delivery, while also helping to mitigate medico-legal risk and potential legal action.

## 1 Introduction

The doctrine of informed consent serves as both a legal safeguard and an ethical cornerstone in contemporary medical practice. It ensures that medical interventions are conducted solely with the patient’s explicit consent, based on a thorough understanding of the clinical circumstances, and forms the ethical foundation of the physician-patient relationship (1–3).

In Italy, the legal framework robustly protects the right to refuse medical treatment. Articles 2, 13, and 32 of the Italian Constitution collectively safeguard the right to make independent decisions regarding one’s integrity and medical treatment. Consent is a prerequisite for any medical intervention, except for specific situations explicitly outlined by law, such as certain public health crises. These provisions establish that medical interventions impacting an individual’s physical integrity require both free and informed consent, reinforcing patient autonomy as a core legal and ethical imperative (4).

The principle of voluntariness in medical treatment is explicitly enshrined in the European Convention on Human Rights and Biomedicine of 1997 (“Oviedo Convention“) (5). Specifically, Article 5 of the Convention asserts: “*An intervention in the health field may only be carried out after the person concerned has given free and informed consent. This person shall be provided with appropriate information regarding the purpose, nature, consequences, and risks of the intervention*” (4, 5).

A shift has occurred in the doctor-patient relationship, where the traditional model of medical paternalism no longer prevails (6, 7). The patient is now regarded as an empowered participant in the decision-making process, making informed choices that align with their personal values and life objectives. The physician maintains the responsibility to ensure that the patient’s choices are both well-informed and aligned with their best interests. This balance requires effective communication, thorough information dissemination, and a commitment to facilitating informed consent.

In Italy, to further institutionalize this patient-centered approach, Law No. 219/2017 “Provisions on informed consent and advance directives,” has been pivotal (8).

Article 1 of this law specifically addresses informed consent, stipulating that: (i) patients must receive clear, comprehensive, and understandable information regarding their diagnosis and available treatment options; (ii) healthcare professionals are required to respect a patient’s decision, even if such refusal may lead to deterioration of their condition or death; (iii) institutions must provide training to staff and establish care pathways that enable informed decision-making (9).

Within the legislative framework, important consideration concerns the adoption of evidence-based clinical practices and the enforcement of patient safety protocols.

These principles are explicitly stipulated in Law No. 24 of March 8, 2017, titled “Provisions on the safety of care and the assisted person, as well as on the professional liability of health professionals” (10). This law emphasizes that the safety of care is a fundamental aspect of the right to health guaranteed to citizens. It is achieved through a multidisciplinary framework of structural, technological, and organizational resources (11, 12).

In this context blood transfusion, while being a vital therapeutic tool, also represents a limited resource within the healthcare system. Even when used appropriately, it still carries inherent risks for the patient, and its availability depends entirely on voluntary donations from citizens.

This limitation led the World Health Organization (WHO), through Resolution WHA63.12 in 2010, to urge all Member States to implement Patient Blood Management (PBM) strategies as a “*vital need to strengthen blood establishments and ensure the quality, safety and efficacy of blood products*” (13).

PBM outlines an evidence-based, multidisciplinary approach to optimizing the care of patients who may require transfusion. This approach minimizes the need for transfusion through measures such as anemia management without transfusion, cell salvage, and the utilization of anti-fibrinolytic drugs to reduce bleeding, as well as restrictive transfusion. By adopting this approach, patients receive the optimal treatment, and avoidable and inappropriate use of blood and blood components is reduced (14).

Additionally, unnecessary transfusions entail heightened risks and associated costs without commensurate benefits (15).

In Italy, the National Blood Centre, in line with this resolution, included the goal of achieving national self-sufficiency among the objectives of the 2012 National Plan for Blood and Blood Component Self-Sufficiency. The Decree of the Italian Ministry of Health dated 4 September 2012, titled “*Program for National Self-Sufficiency of Blood and its Products for the Year 2012*,” established the need to define and implement innovative and more effective methods and measures to ensure adequate clinical and organizational management of blood. In 2015, the Italian Ministerial Decree “*Provisions relating to the quality and safety requirements of blood and blood components*” (Ministerial Decree of 2nd November, 2015 Official Journal of the Italian Republic-series n. 300 of 28th December, 2015) supplements this framework specifically for blood transfusions, mandating written consent or explicit dissent for transfusion products and providing informational materials to patients. In 2016, the Italian National Blood Centre (CNS) published the *“Guidelines for the Patient Blood Management (PBM) Program,”* which include 32 recommendations based on a pragmatic approach to the implementation of a PBM strategy (16–19). The aim is to prevent and significantly reduce the use of blood components and plasma-derived medicinal products by applying the so-called PBM three pillars (13):

1. Optimization of the patient’s erythropoiesis,
2. Minimization of blood loss and bleeding,
3. Optimization of the patient’s physiological reserve to enhance tolerance to anemia.

The document recognized PBM as a multi-professional and multidisciplinary approach. It assessed that the measures recommended by the guidelines would be sustainable, both economically and in terms of transfusion safety. By adopting these measures, the healthcare system could potentially reduce the costs associated with transfusion therapy.

In 2017, the European Commission has delineated PBM as the contemporary paradigm of blood management, encompassing an evidence-driven, multidisciplinary, and multimodal therapeutic approach. This approach facilitates the individualized management and preservation of patients’ blood during surgical and non-surgical interventions (20).

The implementation of PBM programs worldwide has led to significant reductions in transfusions and improved patient outcomes. These systems possess substantial adaptive capacity, responding effectively to deficiencies or heightened demands. It is the paramount responsibility of all clinicians who bear primary accountability for the quality and safety of a patient’s clinical management to guarantee that the patient’s blood is appropriately managed (21).

Therefore, PBM becomes a “standard of care” in patient management in several disciplines, alongside its recognition as a patient safety practice (15, 22).

The current guidelines, consensus documents, and international and national standards underscore that blood and transfusion management is a systematic process. This process, guided by a safety-first approach and a focus on efficacy, enables proactive planning and management of bleeding risks and the necessity for transfusions tailored to individual patients.

This structured approach encounters significant challenges when patients refuse blood transfusions (6, 23). The refusal, often due to religious beliefs, presents complex ethical dilemmas for healthcare providers, requiring a balance between patient autonomy and professional obligations (24–27).

It presents a contemporary challenge to determine whether bloodless surgery achieved through optimal PBM is feasible and safe. Several studies underscored the beneficial effects of bloodless surgical treatment on primary outcomes. These findings demonstrate that blood-sparing surgery is safe when efficient PBM is performed in patients who are unable to receive blood transfusions. A multidisciplinary patient-centered approach including PBM strategies, peri-operative assessment, and advanced surgical techniques may yield positive results in selected patients refusing transfusions (22, 28–30).

In Italy, the regulation of bloodless treatment is based on the general principles of patient autonomy, informed consent, and medical ethics, but also includes specific provisions including Law No. 219/2017 on informed consent and advance directives.

Regarding the informed consent process for the transfusion of blood components and plasma-derived products, Article 24 of the Italian Ministerial Decree of 2015 makes specific reference to the informed consent of the transfusion recipient, explicitly requiring written consent or an express declaration of refusal, always preceded by adequate information (17, 18).

Nevertheless, part of Article 24 is no longer compatible with current legislation where states: “*In cases of imminent danger to life and the onset of unconsciousness in the patient that prevents the acquisition of consent, the physician may proceed with the transfusion of blood even without the patient’s consent. The circumstances constituting the state of necessity must be specified and documented in detail in the medical record*.” This formulation is currently inadequate and needs revision to comply with the provisions set out in Law No. 219/2017 which allows individuals with legal age and full mental capacity to express their wishes in advance through Advance Directives (ADs), including specific decisions on blood transfusion (31).

A national database established by the Italian Ministry of Health collected ADs, promptly updated in the event of renewal, modification or revocation as guaranteed by the Law 219/2017. The patient’s attending physician and the directive’s author may access the database when necessary (32).

Refusing a single part of a comprehensive treatment plan does not mean rejecting the whole approach. Patients must understand that their decision can affect the quality of healthcare, including its effectiveness, risks, and safety, and may affect their health or wellbeing. Conversely, healthcare providers must consider patients’ wishes, but a competent and informed patient accepts the clinical risks without imposing civil or legal liability on the medical professionals. However, if a physician thinks a patient’s request is impractical, due to high risk or therapeutic failure, they are not ethically or professionally obliged to comply, especially when it contradicts established clinical standards or ethical principles (33, 34).

In this context, it becomes essential to ensure the development of a transparent, reproducible, well-documented, and objective framework for communication and information-sharing.

To help healthcare providers deal with these scenarios, the Authors presents a model based on a retrospective analysis of medico-legal consultations over 5-years period in University Hospital “Città della Salute e della Scienza” in Turin, Italy. This integration seamlessly combines clinical strategies, notably PBM, with robust legal safeguards and well-defined communication protocols.

## 2 Objectives

The primary objective of the study is to develop a structured, PBM-oriented communication process for patients who refuse blood transfusions, in alignment with current legal and ethical standards governing informed consent. This procedure outlines each step of the process, from initial contact with the patient to the final documentation of their choices, analyzing where risks may arise.

The secondary objective is to evaluate the clinical applicability and effectiveness of this approach in routine medical practice.

The proposed model integrates evidence-based clinical strategies, such as PBM, and medico-legal risk mitigation protocols, to optimize the safety, efficacy, and legal defensibility of care delivery. The aim is to standardize and formalize the process of communication and information provided to patients who refuse blood transfusions, within the framework of hospital management practices. Implementing a systematic process helps reduce the risk of delivering inaccurate or incomplete information, while ensuring transparency and traceability in obtaining informed consent (or refusal) for transfusion. This approach also minimizes the likelihood of legal actions against the healthcare institution.

## 3 Materials and methods

The study entailed systematically collecting and analyzing the medico-legal consultations conducted over a 5-year period, from 1 January 2017 to 31 December 2021, at the “AOU Città della Salute e della Scienza di Torino” University Hospital in Turin. Only the consultations regarding adult patients, refusing transfusions in non-emergency settings, as distinct from refusal of treatment, were gathered.

Consultation is a formal opinion provided by a specialist in legal medicine, requested by physicians from other hospital departments. These consultations address clinical management, therapeutic decision-making, and discharge planning for admitted patients (35). Following each consultation, the specialist completes a standardized form, routinely filed in the patient’s medical record, documenting patient details, date and time, requesting department, diagnostic question, and the consultation outcome.

Data extracted included:

- Quality and completeness of documentation.
- Multidisciplinary participation in communication.
- Patient decisional capacity and expression of autonomy, including presence of AD.
- Clinical outcomes following alternative therapeutic pathways with PBM.

The informed consent process was divided into several key phases and sub-phases. Each sub-phase was mapped in terms of activities and responsible professionals.

Terminology is used as follows:

- Referring physician: the physician responsible for the patient’s medical care and for initiating contact with the Legal Medicine Unit to request a consultation.
- Multidisciplinary team: a group of professionals from different specialties, responsible for managing the patient’s care pathway and jointly participating in the decision-making process.
- Healthcare Management: the Director of the Healthcare Department involved in the process.

Risk estimation for each sub-phase was performed through risk assessment matrices with quantitative and qualitative approaches, using the PROSA Lean Risk Assessment software (Exprit S.r.l., Florence, IT; https://prosa.improvequality.it). PROSA is a software tool that provides support for risk assessment and utilizes methods like Strengths, Weaknesses, Opportunities, and Threats (SWOT) analysis for context analysis and Failure Mode and Effects Analysis (FMEA) for process risk analysis.

For each sub-phase of the information process, the authors identified the following elements: context (e.g., quality, patient safety, ethical issue), potential threats/errors, outcomes, underlying causes, and existing control measures. Using risk assessment matrices generated by the software, it was assigned both an impact rating (i.e., severity of harm, ranging from no harm to catastrophic) and a probability rating (i.e., likelihood of occurrence, from improbable to frequent). Each risk was evaluated according to its estimated probability and severity of harm.

The software calculated a preliminary risk score using the formula *R = Probability × Impact*, which was subsequently adjusted based on detectability (from certain to remote). The overall risk level was then classified using a five-tier colorimetric scale developed by the software: light blue (score 0–4, negligible risk), green (score 5–18, low), yellow (score 19–48, relevant), orange (score 49–100, critical), and red (score 101–125, high).

To evaluate the applicability of the proposed communication process within its operational context, a SWOT analysis was elaborated by the software. Numerical values based on the impact assessment was assigned by the software in a range of impact’s severity (grade 1—Minor importance, grade 2—Fairly important, grade 3—Moderately important, grade 4—Very important, grade 5—Critical / Decisive).

**Figure 1:**
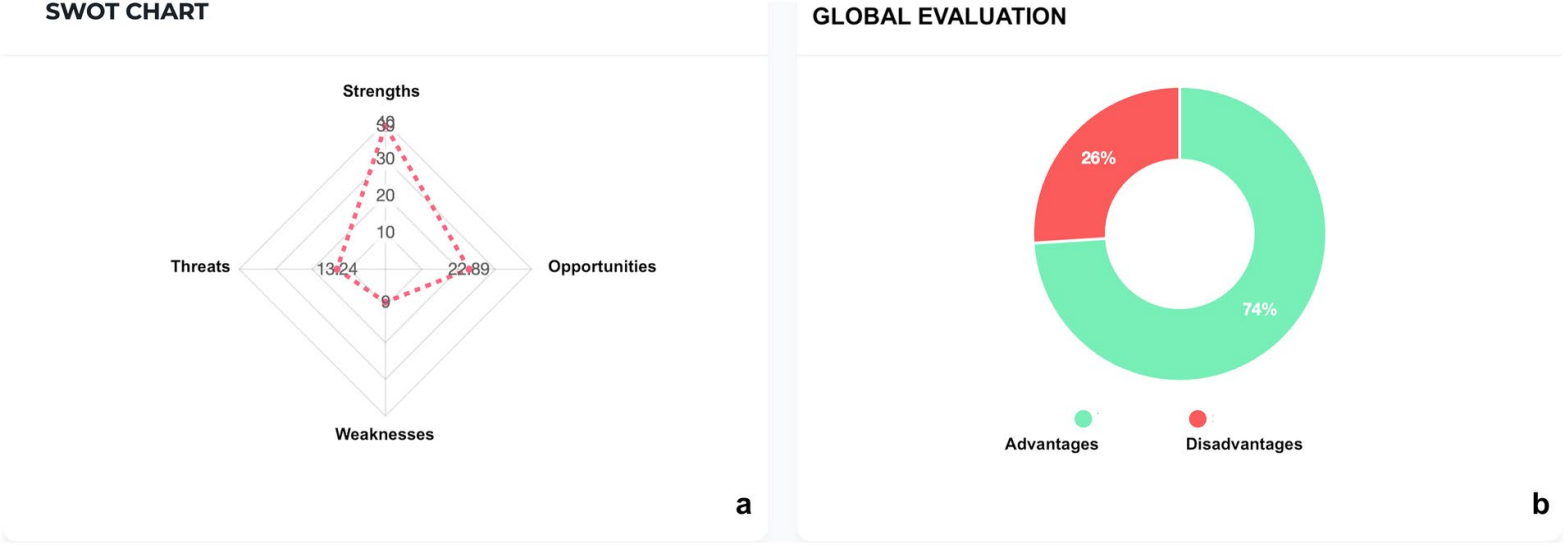
SWOT Analysis of information process: **(a)** SWOT chart, **(b)** global evaluation.

These values are graphically reported in Figures 1a,b, as elaborated by the software.

## 4 Results

The study sample comprised 16 clinical cases undergoing medico-legal consultation for refusal of blood transfusions.

The underlying medical conditions included anemia (37%), cardiovascular surgery (19%), kidney diseases (12%), oncological diseases (13%), trauma (6%), and other conditions (13%).

Consultations were requested by the following departments: critical care medicine (*n* = 4), surgical specialties (*n* = 6), and clinical specialties (*n* = 6).

The patient cohort included 10 male patients (62.5%) and six female patients (37.5%), with mean ages of 65.9 years and 58.3 years, respectively.

### 4.1 Reporting of the consultation

In all cases, the consultation was thoroughly documented and included: date, time, and location of discussions; identification of involved healthcare professionals and presence of family members or patient-appointed representatives; clear statement of diagnosis, proposed treatment, specific risks (including bleeding and probability of transfusion), and consequences of refusal; availability or presentation of administrative documentation, such as previously AD or other declarations provided by the patient during the interview; presentation of alternative therapeutic options, with relative risks and benefits, based on PBM principles; patient decisions formally recorded and signed by all parties.

Family members were present in 10 of the 16 cases. Advance directives were presented in 3 cases.

Following the consultation, two patients were transferred to other facilities, and one procedure was withheld due to excessive risk in the absence of transfusion. Thirteen patients underwent procedures following PBM protocols, with favorable discharge outcomes and without the need for transfusion.

### 4.2 Risk assessment of consultation process

The process was divided into seven phases:

1. Request for consultation and contact with the referring physician and/or the requesting facility.
2. Collection of administrative documentation.
3. First interview with the referring physicians who requested the consultation.
4. Second interview with the multidisciplinary team and review of medical documentation.
5. Third interview with the multidisciplinary team and the patient.
6. Diagnostic–therapeutic resolution by the medical team.
7. Reporting of the interview including the consultation outcome.

The phases were further subdivided into a total of 27 sub-phases as showed in Table 1.

**Table 1:** The medico-legal consultation process subdivided into seven key phases and 27 sub-phases with risk assessment through colorimetric scale.

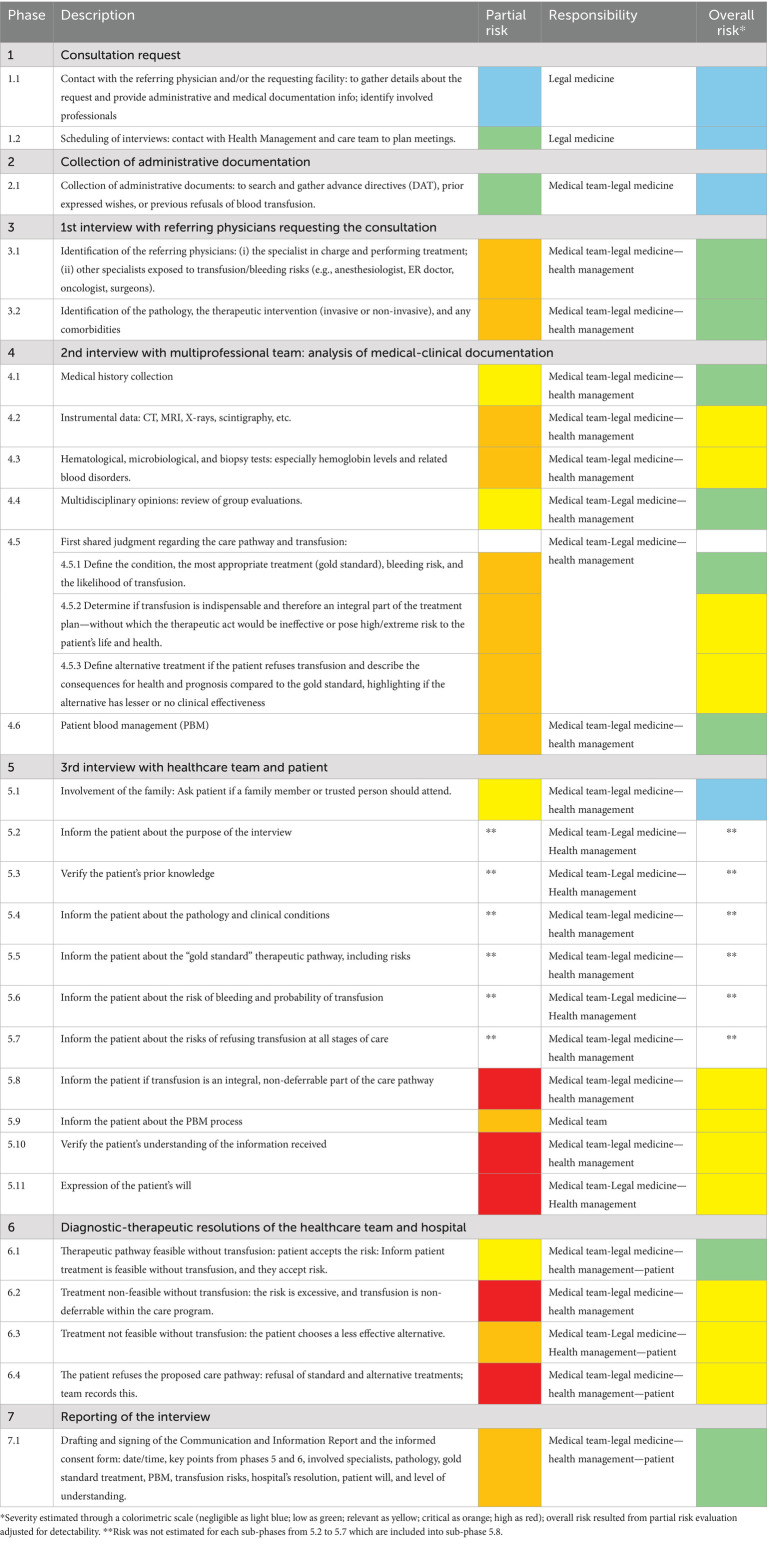

Partial and overall risk assessment, before and after the adjustment for detectability level, was classified according to five-tier colorimetric scales elaborated by the software.

The partial risk was critical or high for:

- Sub-phases 4.2, 4.3, 4.5.2, 4.5.3, 5.9, and 6.3 (critical—orange).
- Sub-phases 5.8, 5.10, 5.11, 6.2 and 6.4 (high—red).

Overall risk assessments showed the following results:

- Negligible risk (light blue) for sub-phases 1.1, 1.2, 2.1, and 5.1.
- Low risk (green) for sub-phases 3.1, 3.2, 4.1, 4.4, 4.5.1, 4.6, 6.1, and 7.1.
- Relevant risk (yellow) for sub-phases 4.2, 4.3, 4.5.2, 4.5.3, 5.8, 5.9, 5.10, 5.11, 6.2, 6.3, and 6.4.

Specific risk was not estimated for each sub-phases from 5.2 to 5.7 which are included into sub-phase 5.8 with relevant risk (orange).

### 4.3 SWOT analysis of the consultation process

The results of the SWOT analysis are presented in Table 2.

**Table 2:** SWOT analysis of the medico-legal consultation process.

Strengths	
Description	Impact*
Presence of legal medicine experts	4—very important
Family involvement	3—moderately important
Written recording	5—decisive
Multidisciplinary team	5—decisive
Option for patient inclusion in PBM	4—very important
Available documentation	5—decisive
Knowledge of regulation framework	4—very important
Active patient involvement	5—decisive element
Preliminary meeting with referring physicians	4—very important

Weaknesses	
Description	Impact
Communication method	2—fairly important
Communication timing	2—fairly important
Preconceived medical positions	2—fairly important
Complexity of the topic	2—fairly important
Recording method	1—minor importance

Opportunities		
Description	Impact	Probability
Definition of the best care pathway	4—very important	99%
Assessment of bleeding risk and probability of transfusion	5—decisive	100%
Patient awareness in health management	5—decisive	90%
Request for active patient involvement in care pathway	5—decisive	100%
Available regulation framework	4—very important	100%

Threats		
Description	Impact	Probability
Incomplete recording of interview	4—very important	10%
Incorrect bleeding risk estimation	4—very important	2%
Lack of patient understanding of the information provided	5—decisive	10%
Incomplete analysis of records	4—very important	2%

*Severity of impact: grade 1—minor importance, grade 2—fairly important, grade 3—moderately important, grade 4—very important, grade 5—critical/decisive.

Strengths related to the communication process in cases of transfusion refusal with very important (grade 4)/ decisive (grade 5) impact included:

- Presence of Legal Medicine experts (grade 4).
- Written recording (grade 5): formal and structured outline of the interview.
- Multidisciplinary team (grade 5): a multi-professional team (clinicians, risk managers, legal and ethics specialists) enhances the quality of information.
- Option for inclusion of the patient in PBM (grade 4): structured alternative to transfusion.
- Availability of documentation (grade 5)
- Knowledge of regulatory framework (grade 4):
- Active patient involvement (grade 5): strengthens shared decision-making.
- Preliminary meeting with the referring physician (grade 4): aligns the multidisciplinary team and prepares the final communication to the patient.

Weaknesses (grade 2 fairly important/grade 1 minor importance) included:

- Methods and timing of communication: risk of delayed or poorly structured interviews.
- Preconceived medical positions: may undermine the neutrality of the consent process.
- Complexity of the topic.
- Recording method: incomplete or ambiguous documentation weakens consent validity.

Opportunities were:

- Definition of the best care pathway by referring physicians (grade 4).
- Assessment of the risk of bleeding and transfusion probability (grade 5) (shared scores/algorithms).
- Patient awareness in health management (grade 5)
- Request for active patient involvement in care pathway (grade 5)
- Availability of regulation framework (grade 4)

Threats included:

- Incomplete recording of the interview (grade 4): medico-legal risk and potential for dispute.
- Error in estimating bleeding risk (grade 4)
- Lack of patient understanding of the information provided (grade 5)
- Incomplete analysis of records by the healthcare team (grade 4): loss of information.

The values of impact’s severity are graphically reported in Figures 1a,b, as elaborated by the software.

The global evaluation revealed a favorable outcome in 74% of aspects, with only 26% exhibiting disadvantages in the adoption of the consultation process.

## 5 Discussion

The ethical and legal implications arising from the refusal of potentially life-saving treatments, such as blood transfusion, are of considerable significance (36, 37). The right to refuse medical treatment is universally recognized as a fundamental principle of personal liberty and must always be respected. In such circumstances, patients must be made aware that their choice carries consequences in terms of the optimal healthcare performance achievable, as well as its associated efficacy, risks, and safety profile (38, 39).

In this challenging context, it becomes imperative to safeguard the patient’s health and safety while demonstrating to the broader community the effectiveness and transparency of healthcare provision.

The process of information exchange and communication should be thoroughly documented and transparent, ensuring that free and informed consent is obtained from a competent individual, without coercion, misinformation, or conflicts of interest (40).

Informed consent for blood transfusions is often inconsistent in many countries due to the lack of mandatory separate consent and limited published audits on patient information and understanding (41).

In USA, the Joint Commission and American Association of Blood Banks (AABB) require informed consent for blood transfusions, including risks, benefits, and treatment alternatives (even nontreatment), the opportunity to ask questions, and the right to accept or not transfusion. Friedman M et al. showed that a small number of patients were reported to be unsure of transfusion indications, highlighting a need for improved healthcare staff education to communicate with the patients (42, 43).

In 2000, the International Society of Blood Transfusion (ISBT) Code of Ethics defined ethical and professional principles for blood transfusion activities and services. However, it is not a detailed “operational” guideline for informed consent in patients but more a set of ethical principles (44).

In 2017 clinical experts developed the “Simplified International Recommendations for the Implementation of Patient Blood Management (SIR4PBM)” where a clear recommendation is that informed consent for allogeneic blood products should be obtained prior to transfusion. A written information form, beside the consent form, should be include benefits, risks, and alternatives according to the ethical principles detailed in the ISBT Code of Ethics (45).

In United Kingdom, Advisory Committee on the Safety of Blood, Tissues and Organs published in 2020 updated guidelines “Patient Consent for Blood Transfusion,” which establish that informed and valid consent must be obtained and documented in clinical records prior to transfusion for all patients in whom transfusion is likely or certain to occur. These guidelines outline the possibility of blood component refusals, including those based on Advanced Directives, and advise on legal aspects of consent, which should be covered by standard hospital practices. However, these recommendations must align with current legislation on consent and relevant regulations. From the previous version in 2011, the SaBTO recommendations emphasize shared decision-making and adequate communication as elements of the consent process, not signed consent. While this is the minimum requirement, organizations may choose to implement signed consent (46).

A study conducted in Australia revealed that hospitals implementing an informed consent protocol for blood and blood product transfusions demonstrate a substantial level of compliance with documentation requirements for transfusion consent. Consequently, these institutions are better positioned to adhere to national guidelines and accreditation standards (47).

Also, medical trainees and physicians often receive inadequate and inconsistent training in obtaining informed consent for blood transfusions, leading to consent that is sometimes incomplete or poorly understood. Existing guidelines outline required content but lack practical tools like sample narratives or content prioritization (48).

This study examines the communication and informed consent process during medico-legal consultations in cases of transfusion refusal, using a structured risk assessment analysis within the operational context of a Legal Medicine Unit. The authors propose a step-by-step framework to support clinical practice and help ensure that the essential elements of informed consent for transfusion are consistently addressed.

Process-oriented and risk management approaches represent indispensable tools within the current framework of patient safety. These frameworks should encourage healthcare institutions to adapt shared protocols to their specific contexts, developed collaboratively with healthcare professionals and the patient. Such strategies facilitate the provision of effective, patient-centered care while ensuring that outcomes are verifiable and traceable.

The analysis of the clinical cases facilitated a comprehensive evaluation of both procedural strengths and vulnerabilities of the process.

Most activities were classified as negligible or low risk, particularly those involving the identification of specialists, the review of medical documentation, and the preliminary multidisciplinary discussion of the patient’s case. These findings suggest that a well-structured procedural approach, supported by clinical experience and multidisciplinary input, can significantly reduce medico-legal exposure in high-sensitivity scenarios such as transfusion refusal.

Nevertheless, certain sub-phases emerged as critical points. Relevant risks were identified in the analysis of diagnostic results (radiological, laboratory, microbiological, histopathological) and in the initial shared decision regarding the necessity of transfusion. In these contexts, misinterpretation or incomplete review could result in inappropriate therapeutic decisions, potentially leading to patient harm and legal claims.

Notably, high risk ratings were assigned to sub-phases concerning the communication of transfusion necessity and the feasibility of proposed PBM strategies, as well as to patient decisions rejecting the standard-of-care pathway. From a medico-legal perspective, these phases are pivotal: failure to ensure accurate, comprehensible, and complete information transfer at these junctures can invalidate consent, undermine patient autonomy, and expose healthcare institutions to litigation.

The SWOT analysis confirmed the operational applicability of the process in the current setting, highlighting its strengths in structural and organizational aspects.

The presence of Legal Medicine experts, the use of formalized documentation, and the integration of a multidisciplinary team were identified as pivotal factors in ensuring the quality of communication and compliance with current regulation.

The inclusion of PBM as a structured alternative to transfusion was recognized as both a clinical and legal safeguard, aligning with contemporary best practices and patient safety.

However, weaknesses were also apparent. Variability in communication methods and timing, potential bias in clinical positions, the inherent complexity of the subject matter, and occasional lapses in documentation quality represent areas for enhancement. The health literacy gap can emerge as a barrier to truly informed decision-making, necessitating targeted interventions such as plain-language materials and structured teach-back techniques.

Opportunities with substantial impact included the delineation of optimal non-transfusion treatment pathways, the adoption of standardized bleeding risk assessment tools, and systematic patient engagement in care planning. While threats, although generally of low probability, carried high potential impact—most notably incomplete interview documentation, errors in bleeding risk estimation, and insufficient review of clinical records—they should not be overlooked.

The global evaluation of the presented process revealed a 74% advantage in terms of positive aspects, while only 26% presented disadvantages in adopting this method.

The combined findings of the risk assessment and SWOT analysis support a proactive, continuous improvement approach to managing transfusion refusal cases.

In summary, priority actions of the approach to the patient refusing blood transfusion should include:

- The standardization of interview procedures, enhancing staff training in communication neutrality, embedding PBM pathways by default when appropriate, and conducting regular audits to monitor both documentation quality and patient comprehension;
- Verification of patient understanding (teach-back): the patient (and, if consenting, the family) should repeat the key points in their own words;
- Forms and documentation: use clear, standardized forms with mandatory fields (e.g., non-transfusion alternatives, risks/benefits, patient preferences);
- Bleeding risk stratification: apply shared scoring systems or protocols and document them in the medical record; obtaining a second opinion for high-risk cases can be useful;
- PBM pathways: it must be activated “by default” when appropriate, ensuring timely referral;
- Healthcare staff training, including skills for communicating bad news, maintaining neutrality during the interview, and recognizing cognitive or emotional bias;
- Audit and feedback: conduct random audits on the completeness of documentation and the rate of documented patient understanding; monitor both process and outcome indicators.

Although the SWOT analysis is inherently constrained by its qualitative nature, its integration with quantitative risk assessment provides a robust decision-making framework. By concentrating improvement efforts on the sub-phases exhibiting the highest concentration of weaknesses and threats, the process can be incrementally optimized, thereby augmenting the proportion of advantages over disadvantages in its application.

The overall analysis can allow hospitals and healthcare professionals to plan and manage interviews with patients, ensuring that they can prevent and control any potential threats to the patient’s care and the actions of the healthcare professionals involved. It also aims to prevent medical-legal litigation that could arise from inadequate communication or information to the patient, or from a lack of inadequacy of interview drafting. The violation of a patient’s right to self-determination itself constitutes a compensable form of damage under Italian law.

The main limitation of the study is the small number of medico-legal consultations, which is due to the fact that the Legal Medicine Unit has only become fully operational in the Hospital in recent years. Consequently, the findings can only be used to describe and evaluate the relevance of the information process conducted with forensic medicine within a representative Italian hospital.

## 6 Conclusion

PBM is a patient-centered approach to managing blood loss, emphasizing diagnosis, patient-specific therapeutic options, and patient safety as a legal requirement that must be implemented by all facilities and for all patients (49, 50). PBM encompasses more than the optimization of transfusion practices. Beyond correcting iron deficiency and anemia, the three fundamental pillars integrate additional approaches aimed at preserving and managing the patient’s own blood, expecting to further decrease the need for perioperative transfusions (51, 52). Several Authors demonstrated the evidence base on PBM regarding its feasibility, and impact in clinical practice by demonstrating cost-effectiveness in the aim to integrate PBM into routine practice (53, 54).

Its application involves several issues, including the selection of patient-specific therapeutic strategies tailored to the underlying diagnosis, active patient involvement in shared decision-making, the acquisition of informed consent, and the provision of continuous clinical follow-up (50).

Informed refusal of transfusion, as distinguished from refusal of care, constitutes a legally protected right (23, 26, 55). Healthcare providers and institutions cannot passively acquiesce to such decisions. Rather, they must actively manage the associated risks through structured communication, multidisciplinary decision-making, and meticulous documentation—challenges that can only be effectively addressed through a process-oriented approach, which ensures that the entire informational pathway with the patient is documentable, traceable, and transparent.

This approach not only complies with the constitutional requirement of informed consent but also mitigates the potential for subsequent medico-legal disputes.

Ultimately, the proposed process offers a replicable framework that can be adapted to other clinical contexts where high-stakes decision-making intersects with patient rights, supporting a culture of safety, transparency, and continuous improvement in healthcare delivery, while also helping to mitigate medico-legal risk and potential legal action.

## Data Availability

The original contributions presented in the study are included in the article/supplementary material, further inquiries can be directed to the corresponding author.
